# Classification of tuberculosis-related programmed cell death-related patient subgroups and associated immune cell profiling

**DOI:** 10.3389/fimmu.2023.1159713

**Published:** 2023-05-02

**Authors:** Jie Shen, Chao Zhao, Hong Zhang, Peipei Zhou, Zhenpeng Li

**Affiliations:** ^1^ School of Medical Laboratory, Weifang Medical University, Weifang, China; ^2^ Office of Academic Affairs, Weifang Medical University, Weifang, China; ^3^ School of Public Health, Weifang Medical University, Weifang, China

**Keywords:** programmed cell death, tuberculosis, immune cell enrichment, single cell RNA-seq, machine learning, biomarkers

## Abstract

**Background:**

Tuberculosis (TB) is the deadliest communicable disease in the world with the exception of the ongoing COVID-19 pandemic. Programmed cell death (PCD) patterns play key roles in the development and progression of many disease states such that they may offer value as effective biomarkers or therapeutic targets that can aid in identifying and treating TB patients.

**Materials and methods:**

The Gene Expression Omnibus (GEO) was used to gather TB-related datasets after which immune cell profiles in these data were analyzed to examine the potential TB-related loss of immune homeostasis. Profiling of differentially expressed PCD-related genes was performed, after which candidate hub PCD-associated genes were selected via a machine learning approach. TB patients were then stratified into two subsets based on the expression of PCD-related genes via consensus clustering. The potential roles of these PCD-associated genes in other TB-related diseases were further examined.

**Results:**

In total, 14 PCD-related differentially expressed genes (DEGs) were identified and highly expressed in TB patient samples and significantly correlated with the abundance of many immune cell types. Machine learning algorithms enabled the selection of seven hub PCD-related genes that were used to establish PCD-associated patient subgroups, followed by the validation of these subgroups in independent datasets. These findings, together with GSVA results, indicated that immune-related pathways were significantly enriched in TB patients exhibiting high levels of PCD-related gene expression, whereas metabolic pathways were significantly enriched in the other patient group. Single cell RNA-seq (scRNA-seq) further highlighted significant differences in the immune status of these different TB patient samples. Furthermore, we used CMap to predict five potential drugs for TB-related diseases.

**Conclusion:**

These results highlight clear enrichment of PCD-related gene expression in TB patients and suggest that this PCD activity is closely associated with immune cell abundance. This thus indicates that PCD may play a role in TB progression through the induction or dysregulation of an immune response. These findings provide a foundation for further research aimed at clarifying the molecular drivers of TB, the selection of appropriate diagnostic biomarkers, and the design of novel therapeutic interventions aimed at treating this deadly infectious disease.

## Introduction

Tuberculosis (TB) is among the deadliest forms of chronic infectious disease in the world, with an estimated 10.6 million affected individuals and 1.4 million TB-related deaths in 2021 alone (https://www.who.int/teams/global-tuberculosis-programme/tb-reports/global-tuberculosis-report-2022). Despite extensive ongoing international efforts, an estimated 1.5 million individuals are expected to die annually of TB, caused by *Mycobacterium tuberculosis* (Mtb), through the year 2030 (1). Prior to the COVID-19 pandemic, TB was the deadliest infectious disease in the world, causing higher levels of mortality than HIV/AIDS (2). Strikingly, up to 25% of the global population is infected with the infectious Mtb pathogen, highlighting a clear need for further transcriptomic studies seeking to elucidate the molecular basis for morbidity and mortality in affected individuals (3–6). Given the lack of effective treatment options for most TB cases, there is a pressing need to better improve patient prognostic outcomes, and the design of more reliable models has the potential to better facilitate targeted therapeutic interventional efforts (7, 8).

Programmed cell death (PCD) is an umbrella term that refers to a range of processes including apoptosis, pyroptosis, ferroptosis, and other less well-studied mechanisms such as alkaliptosis or oxeiptosis (9). Apoptotic cell death has been studied in detail and consists of the spontaneous, ordered death of cells through mechanisms controlled by particular regulatory pathways (10). While traditionally regarded as an unregulated process, a growing body of evidence suggests that necrotic cell death is also inducible and can be maintained through specific mechanisms (11). Pyroptotic cell death is a particularly caustic form of PCD that results in the activation of a robust inflammatory response (12). The ferroptotic and cuproptotic cell death processes are respectively iron- and copper-dependent, and have been tied to a range of diseases (13, 14). Entotic cell death is related to active living cell invasion (15). The release of neutrophil extracellular traps (NETs) has been linked to so-called netotic cell death (16), while excessive PARP-1 nuclease activity can result in the form of PCD known as parthanatos (17). Lysosome-dependent cell death is controlled by hydrolase activity, which increases in the cytosol following the penetration or permeabilization of cellular membranes (18). Autophagy-dependent cell death is a multi-stage regulated process through which lysosomal degradation is tied to mechanisms associated with nutrient cycling and metabolic adaptation (19). Alkaliptosis is a recently defined form of PCD related to alkalization (20), while oxeiptosis is a PCD subtype that is induced through mechanisms related to the ability of KEAP1 to serve as a sensor of oxidative stress (21). The advancement of PCD-related research has led to the development and clinical implementation of a growing number of pharmacological agents associated with these disparate mechanisms.

PCD has been firmly established as an important mediator of the pathogenesis of a range of conditions including specific autoimmune diseases, cancers, neurodegenerative diseases, immunodeficiencies, and developmental disorders (22–24), with analyses of these genes thus providing opportunities for prognostic assessment and targeted therapeutic interventions. Comprehensive details regarding the association between PCD and the pathogenesis of TB, however, are still lacking such that further research is warranted fully documenting the roles of particular PCD-related genes and pathways in individuals infected by Mtb. As such, in the present study a series of machine learning tools were employed to ultimately select seven key PCD-related genes that were associated with TB. The expression patterns of these genes were then used to group TB patients into two subsets, and immune cell abundance was then compared between these groups of patients. Other TB-related diseases were additionally explored, and the association between PCD and immune cell abundance was examined in detail to gain novel insight regarding the molecular basis for the pathogenesis of TB. Together, these results provide new opportunities to more reliably develop diagnostic or therapeutic regimens that can be leveraged to improve TB patient outcomes.

## Materials and methods

### Dataset selection and PCD-related gene identification

The TB-related datasets used for the present study (GSE83456 (25), GSE28623 (26), GSE62525 (27), GSE157657 (28), GSE93272 (29), GSE162635 (30), GSE47460 (31), GSE130499 (32), GSE166253 (33), GSE31210 (34), GSE50772 (35)) were downloaded from the NCBI Gene Expression Omnibus (GEO; https://www.ncbi.nlm.nih.gov/geo/) database. PCD-related genes were collected from GSEA gene sets, KEGG, review articles, manual collation (9) and are shown in **Table S1**.

### Differentially expressed gene identification and analysis

The R “limma” package was used for DEGs identification based on the following cut-off criteria: P < 0.05, |log 2(fold change [FC]) > 1|. DEGs were arranged in volcano plots and heat maps, and were subjected to Gene Ontology (GO) and KEGG enrichment analyses performed using the DAVID online tool (ncifcrf.gov). The R “GSVA” and “GSEABase” packages were used for GSVA analyses assessing different biological functions among clusters with “h.all.v7.5.1.symbols”, “c2.cp. Reactome.v7.5.1.symbols”, and “c2.cp.kegg.v7.5.1.symbols”.

### Immune cell enrichment analyses

Gene expression data and LM22 files were used with the R CIBERSORT algorithm to evaluate immune cell abundance in individual samples (36).

### Single-cell data preprocessing, gene expression quantification and cell-type determination

Raw sequencing reads were obtained from NCBI Short Read Archive (SRA) with the accession numbers SRR11038989, SRR11038990, and SRR11038994 (37). Raw sequencing reads were processed by Cell Ranger (6.1.2) and aligned to the human reference genome (GRCh38). The unique molecular identifier (UMI) count matrices were then imported into the “Seurat” R package. Cells expressing <200 or >3500 genes or a high mitochondrial transcript ratio (> 0.07) were removed. For the remaining cells, NormalizedData, Seurat indVariableFeatures, ScaleData, JackStraw, and FindNeighbors in the Seruat package were used for processing. The principal component analysis (PCA) was performed, and the dimensionality reduction cells were represented by Uniform Manifold Approximation and Projection (UMAP), and the clusters was identified and annotated according to the marker gene composition. The marker genes comes from previous studies (37, 38).

### Machine learning analyses

A Least Absolute Shrinkage and Selection Operator (LASSO) regression approach was employed to improve the regularity, interpretability, and predictive accuracy of predictive models and to select associated variables for model incorporation (39). For these analyses, a support vector machine (SVM) method was implemented which allows for the establishment of a threshold between categories such that sample labeling predictions can be performed based on one or more feature vectors (40). Random forest (RF) approaches, which enable high levels of accuracy, specificity, and sensitivity without being limited by variable conditions, were employed to predict continuous variables without major fluctuations (41). The eXtreme Gradient Boosting (Xgboost) ensemble learning algorithm utilizes decision trees as base learners (42). These LASSO, SVM-RFE, RF, and Xgboost machine learning analyses were implemented using the R “glmnet”, “kernlab”, “randomForest”, and “xgboost” packages. For RF and Xgboost , we selected the top 10 genes in terms of ranking. Intersecting genes among these analyses, as identified with the R “circle” package, were considered to represent hub PCD-related genes.

### Construction and validation of the nomogram

The R “rms” package was used to establish a diagnostic nomogram for TB< while calibration plots and decision curve analyses (DCA) were conducted using the “rmda” and “caret” packages in R. The “pROC” R package was used to evaluate the predictive capabilities of the established model using receiver operating characteristic (ROC) curves.

### Subclustering analyses

Subclusters of TB patients in the analyzed datasets were identified via consensus clustering based on hub PCD-related gene expression using the R “ConsensusClusterPlus” package using the following settings: maxK = 9, clusterAlg = pam, distance = euclidean.

### Quantitative reverse transcription polymerase chain reaction

Seven hub PCD-related genes were further identified for validation. A total of 20 subjects were recruited in this study, including 10 TB patients from Weifang Second People’s Hospital, and 10 healthy volunteers. All patients gave informed consent before the start of the study.

Samples were collected from each participant prior to initial treatment. Total RNA was then extracted from each sample with TRIzol (Invitrogen). Reverse transcription was performed using the HiScript III RT SuperMix for qPCR (+gDNA wiper) (Vazume). Next, qPCR was performed using ChamQ Universal SYBR qPCR Master Mix (Vazume) based on LightCycler^®^ 480 II Real-Time PCR System (Roche). GAPDH served as an internal control. The 2^-ΔΔCt^ method was used to determine the relative expression between TB and HCs for each selected hub genes. The primer sequences used in this study are listed in **Table S2**.

### Identification of potential therapeutic compounds

The connectivity map (CMap) is based on the relationship between genes or drugs to discover potentially effective molecules for certain diseases (https://clue.io/) (43). Through this database, we used the CMap tool in the “query” module through the L1000 platform to identify the DEG, 100 up-regulated and 100 down-regulated genes (select the appropriate number if the number of genes is insufficient) between the high PCD related gene expression group and the low expression group as effective genes, and selected the five potential therapeutic compounds for this disease with the highest CMap score for each disease, Five compounds with the lowest enrichment fraction ≤ 0 were selected as candidate inhibitors.

### Statistical analysis

R v4.2.2 was used for all statistical testing. Figure panels were pieced together by Adobe Illustrator (CC 2020). The significance of the correlation between the two groups was tested by Spearman’s correlation analysis. Data were compared using Student’s t-tests or Wilcox tests, with P < 0.05 as the significance threshold. Visualization of data was performed with GraphPad Prism v.9.5 and R v4.2.2.

## Results

### Differentially expressed gene identification

**Figure 1:**
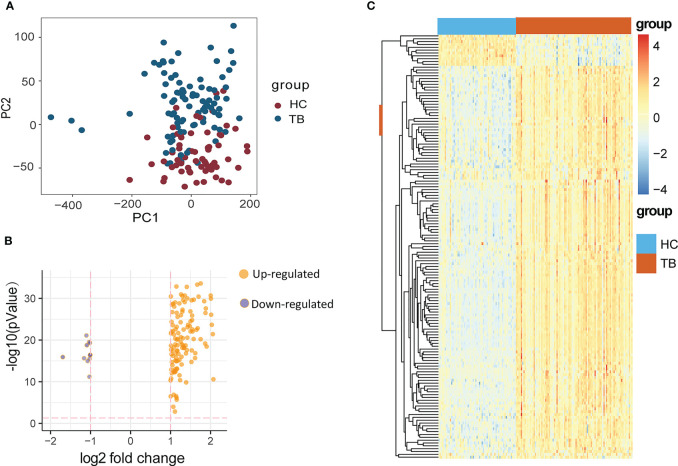
**(A)** PCA diagram showing the distribution of gene expression in HC and TB samples. **(B, C)** DEGs identified between HCs and TB patients were compared with a volcano plot **(B)** and a heat map **(C)**. DEGs, differentially expressed genes; HC, Healthy control; TB, Tuberculosis.

The GSE83456 dataset consisting of 61 healthy control (HC) individuals and 92 TB patients was obtained from the GEO database. A PCA plot (**Figure 1A**) revealed clear differences in gene expression between HC and TB samples, including 138 and 11 genes that were respectively up- and downregulated (P < 0.05, |log2(FC) > 1|). These DEGs were arranged into volcano plots (**Figure 1B**) and heat maps (**Figure 1C**) for visualization purposes.

### Functional enrichment analyses

**Figure 2:**
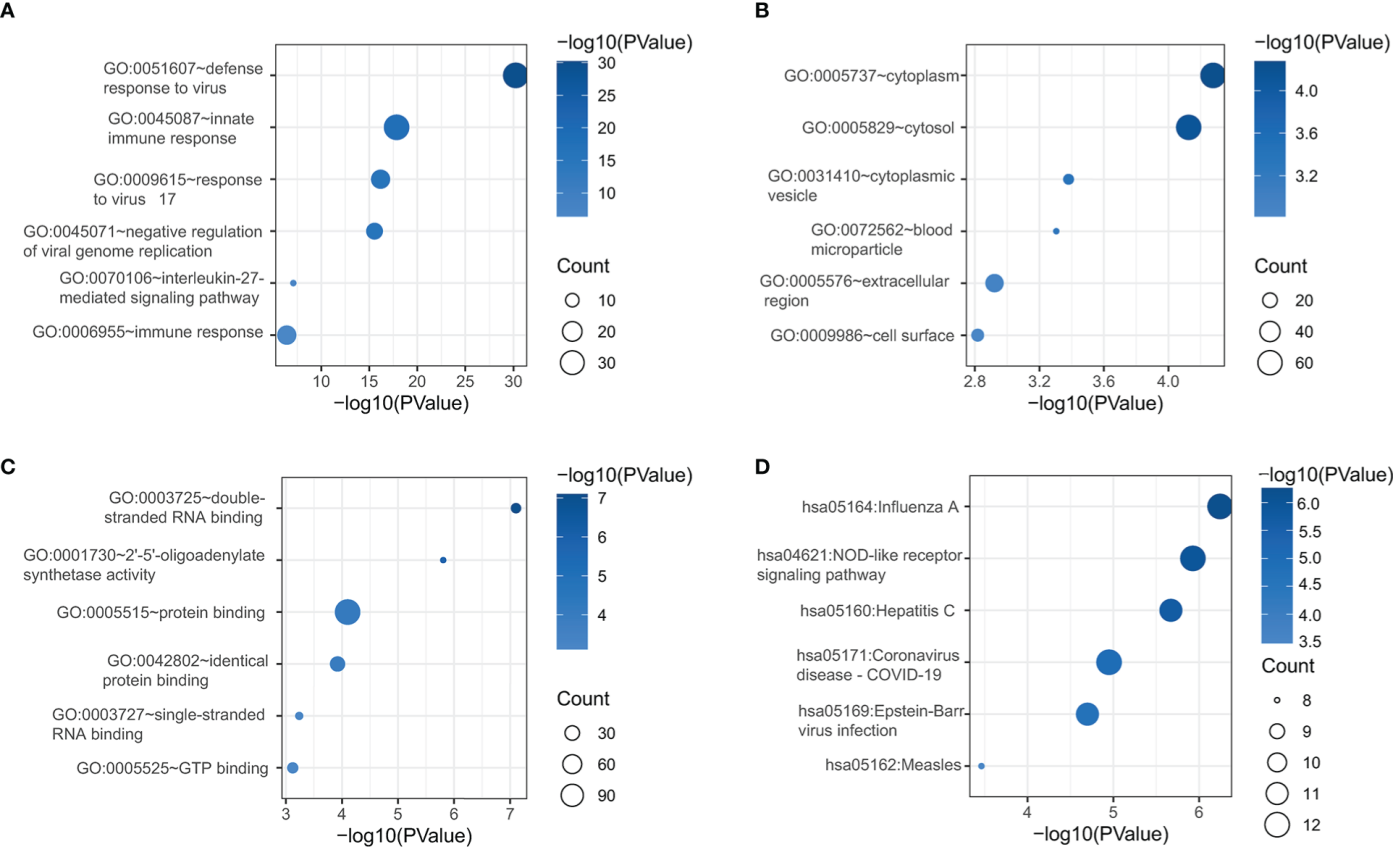
DEG functional enrichment analyses of GO BP **(A)**, GO CC **(B)**, GO MF **(C)**, and KEGG pathway terms **(D)**. GO, Gene Ontology; BP, biological process; CC, cellular component; MF, Molecular function; KEGG, Kyoto Encyclopedia of Genes and Genomes.

The DAVID online tool was next used for GO and KEGG enrichment analyses of identified DEGs. These genes were enriched in GO biological process terms including the following: defense response to virus, innate immune response, response to virus 17, negative regulation of viral genome replication, interleukin-27 mediated signaling pathway, and immune response were enriched (**Figure 2A**). They were also enriched in the GO cellular component terms cytoplasm, cytosol, cytoplasmic vesicle, blood microparticle, extracellular region, and cell surface (**Figure 2B**), as well as the GO molecular function terms double-stranded RNA binding, 2’-5’-oligoadenylate synthetase activity, protein binding, identical protein binding, single-stranded RNA binding, and GTP binding were discovered (**Figure 2C**). These DEGs were also enriched in the Influenza A, NOD-like receptor signaling pathway, hepatitis C, coronavirus disease-COVID-19, Epstein-Barr virus infection, and measles KEGG pathways (**Figure 2D**).

### Immune cell enrichment analyses

**Figure 3:**
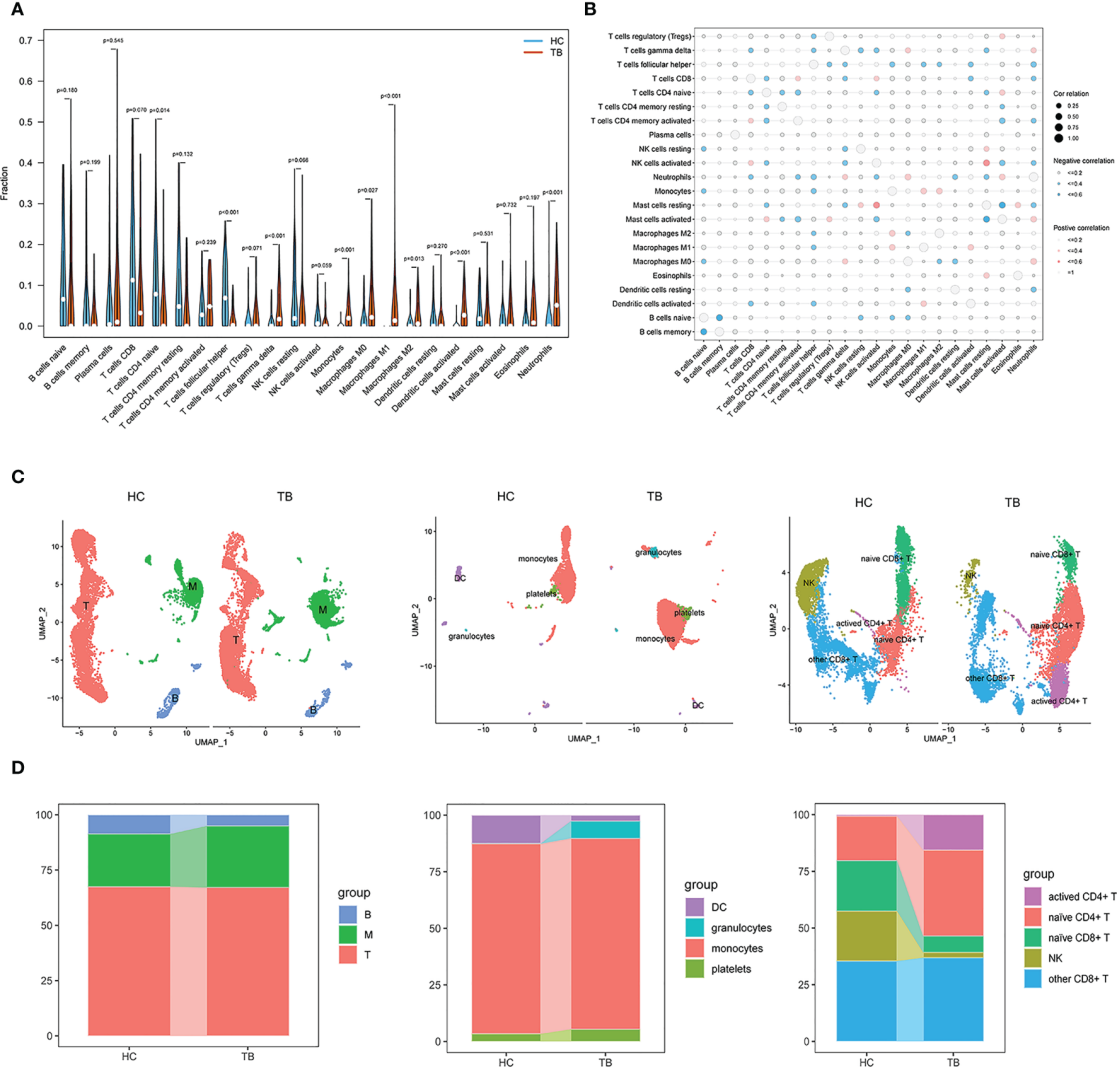
Immune cell enrichment in patients with TB. **(A)** Comparison of 22 immune cell subtypes between patients with TB and HCs. **(B)** Correlations among all 22 analyzed immune cell subtypes. Size and coloration of the circles are proportional to the corresponding Pearson correlation coefficients. **(C)** UMAP visualization of major cell types across three groups (left), Myeloid clusters (middle), and T cells (right). **(D)** Proportion of each defined cell type across major groups (left), Myeloid clusters (middle), and T cells (right). B, B cells; T, T cells; M, Myeloid. NK, Naturel killer.

Given that the majority of the enriched terms identified above were associated with immunological function, this strongly suggested a role for immune function in the pathogenesis of TB. Accordingly, immune cell enrichment analyses were used to compare HC and TB samples. This approach revealed that TB patients exhibited higher levels of gamma delta T cell, monocyte, M0 macrophage, M1 macrophage, M2 macrophage, activated dendritic cell (DC), and neutrophil abundance, whereas they exhibited fewer naïve CD4+ T cells and follicular helper T (Tfh) cells relative to HC samples (**Figure 3A**). Correlation analyses for 22 immune cell subtypes revealed strong negative correlations between naïve B cells and memory B cells, Tfh cells and neutrophils, naïve CD4+ T cells and activated memory CD4+ T cells, activated mast cells and activated NK cells, and resting mast cells and activated memory CD4+ T cells. In contrast, M1 macrophages and activated DCs, M0 macrophages and neutrophils, activated mast cells and neutrophils, resting mast cells and activated NK cells, and resting mast cells, monocytes, and eosinophils were strongly positively correlated (**Figure 3B**). Targeting these immune cell types may aid in the identification of viable therapeutic targets for TB.

We also performed scRNA-seq analysis on peripheral blood mononuclear cells (PBMCs) derived from two individuals, including HC and TB. In total, 20880 cells were taken into analysis (10373 cells from HC and 10507 cells from TB). As shown in **Figures 3C**, **S1**, three clusters were identified, including myeloid cells expressing S100A9, S100A8, S100A12, CD14 and LYZ, T cells expressing CD3D, CD3E, IL32 and CD2, and B cells expressing CD79A, CD79B and MS4A1. We found higher frequencies of myeloid and a lower frequency of B cells in TB compared to HC, which were consistent with the Immune cell enrichment results. Subsequently, the myeloid cells cluster mainly included DC, monocytes, platelets (**Figures 3C**, **S2**) and granulocytes clusters and T cells were marked by NK, other CD8+ T cells, naïve CD4+ T cells and activated CD4+ T cell clusters (**Figures 3C**, **S3**). As shown in **Figure 3D**, monocytes, platelets, granulocytes and activated CD4+ T cells had higher frequencies and NK cells and naïve CD8+ T cells had lower frequency, which were also consistent with the Immune cell enrichment results. Howerer, naïve CD4+ T cells and DC had an inconsistent results. These findings strongly underline the significant role of immune cells in TB development and were further confirmed by additional single cell data analysis (**Figure S4**).

### Hub PCD identification

**Figure 4:**
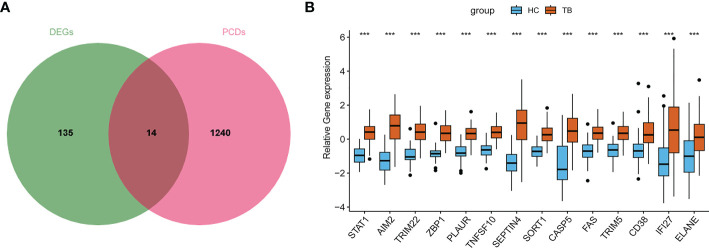
Screening of PCD-related genes in TB. **(A)** The overlap between DEGs and PCD-related genes. **(B)** Overall expression levels for PCD-related genes in TB patients.

Next, fourteen PCR-related DEGs (*STAT1*, *AIM2*, *TRIM22*, *ZBP1*, *PLAUR*, *TNFSF10*, *SEPTIN4*, *SORT1*, *CASP5*, *FAS*, *TRIM5*, *CD38, IFI27*, and *ELANE*) were identified based on the intersection between 149 DEGs and 1254 PCD-related genes (**Figures 4A, B**).

**Figure 5:**
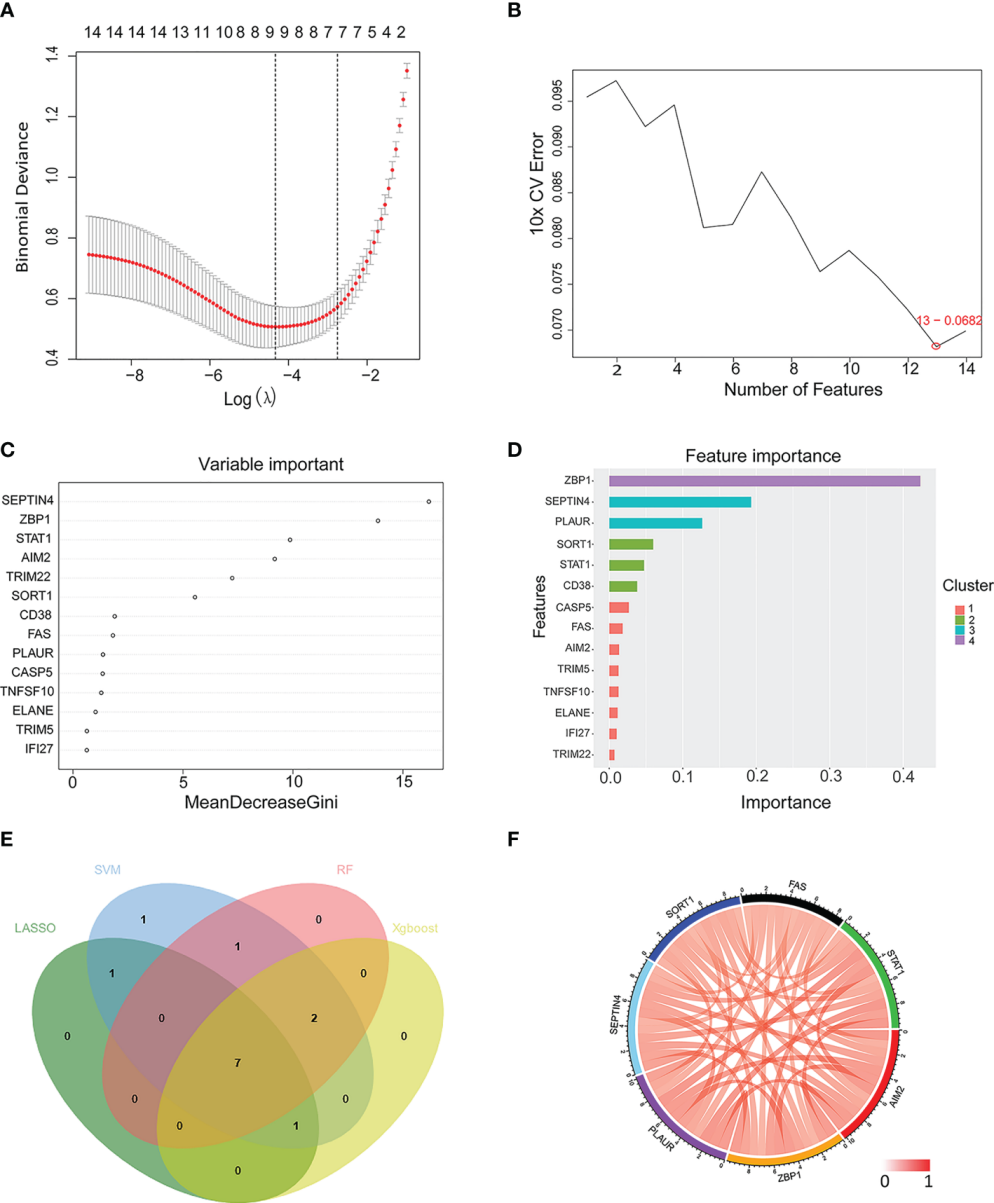
Machine learning-based selection of a PCD-related gene signature. **(A–D)** Use of LASSO regression, SVM, RF, and Xgboost approaches to construct a PCD-related gene signature associated with TB. **(E)** A Venn diagram highlighting overlap among candidate genes identified with these four machine learning algorithms. **(F)** A Circos plot displaying the relationship between the overlapping PCD-related genes. LASSO, least absolute shrinkage and selection operator; SVM, support vector machine; RF, random forest; Xgboost, eXtreme Gradient Boosting.

A LASSO regression analysis, SVM-RFE, random forest, and Xgboost machine learning approaches were next used to construct a seven PCD-related gene signature (**Figures 5A–E**). Three of these genes (*FAS*, *SEPTIN4*, *PLAUR*) were apoptosis-related, while two (*ZBP1* and *STAT1*) were necroptosis-related, one (*AIM2*) was pyroptosis-related, and one (*SORT1*) was lysosome-dependent cell death-related. **Figure 5F** presents correlations among these hub genes.

**Figure 6:**
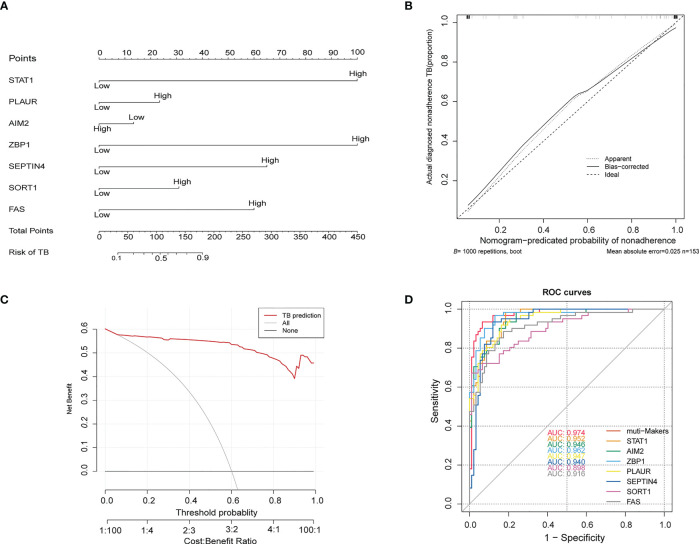
**(A)** Construction of a nomogram model based on seven PCD-related genes. **(B)** Calibration plot assessing the robustness of nomogram predictions. **(C)** Decision curve analysis for the established nomogram. **(D)** ROC curve of the PCD-related signature when used for the diagnosis of TB. ROC, receiver operating characteristic.

These genes were further used to construct a nomogram (**Figures 6A, B**), which exhibited a C-index value of 0.975 (95% CI: 0.952-0.998). In a DCA analysis with a threshold of 0.06–1, this model offered good clinical benefit (**Figure 6C**), and its value was further confirmed through ROC analyses (**Figure 6D**). These findings thus highlighted the excellent diagnostic utility of these seven PCD-related hub genes.

### Associations between PCD-related genes and immune cell enrichment

**Figure 7:**
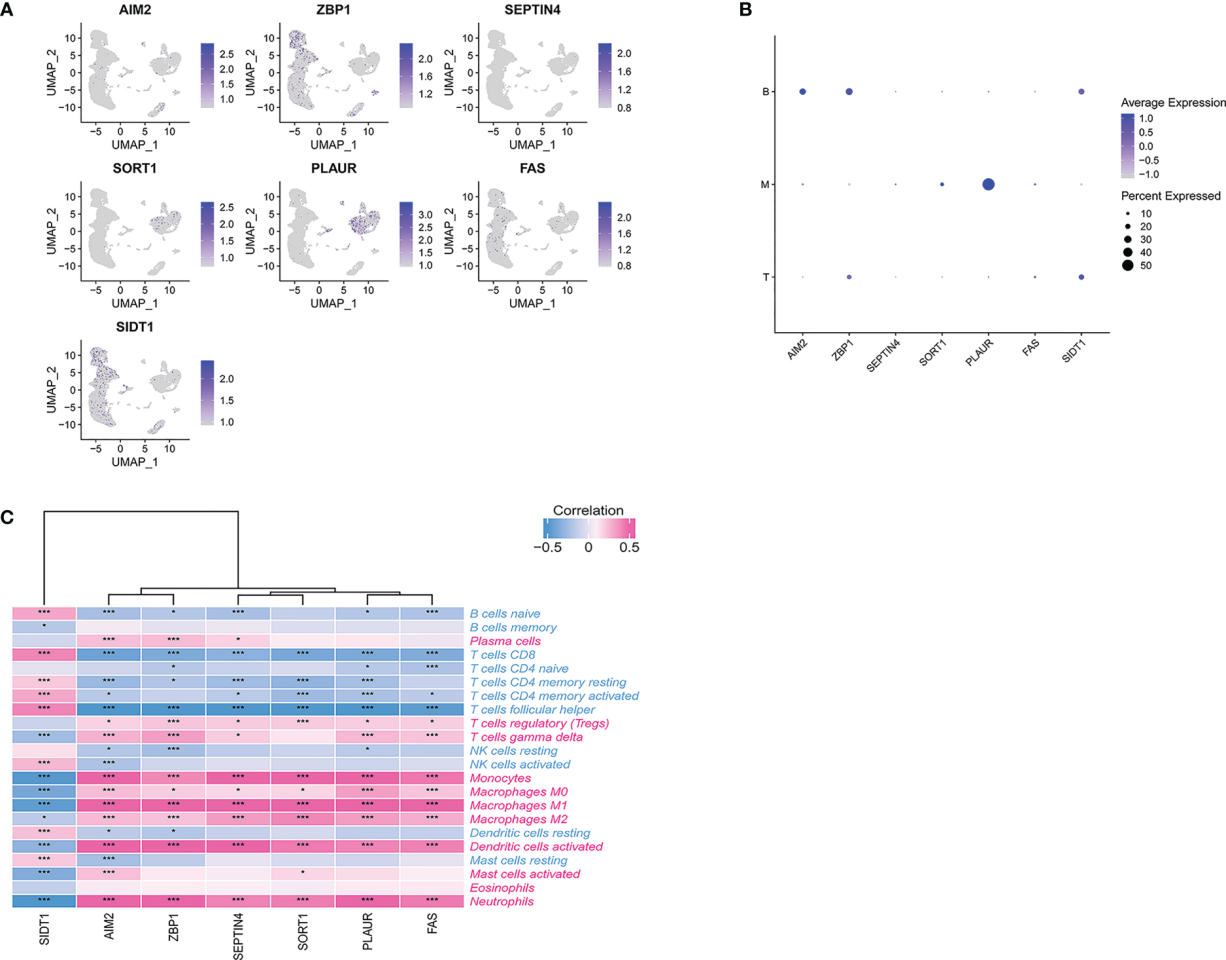
**(A)** scRNA-seq analysis of the expression of PCD-related genes. **(B)** Dot plot showing the PCD-related genes in each cell type. **(C)** Correlations between immune cell enrichment and seven PCD-related genes. *p < 0.05, ***p < 0.01.

We utilized scRNA-seq to analysis the expression level and location of PCD-related genes. As shown in **Figures 7A, B**, *ZBP1* and *SIDT1* were dominantly expressed in T and B cells. *PLAUR* and *SORT1* were primarily expressed in myeloid cells. *AIM2* was enriched in B cells, while *FAS* and *SEPTIN4* showed lower expression in all cells.

To more fully understand the relationships between these hub PCD-related genes and immune cell enrichment, further correlation analyses were conducted. This approach revealed a strong negative association between six of these PCD-related genes (*AIM2*, *ZBP1*, *SEPTIN4*, *SORT1*, *PLAUR*, *FAS*) and the levels of Tfh cells and CD8+ T cells (**Figure 7C**).

These same six genes were also significantly positively correlated with the abundance of neutrophils, activated DCs, monocytes, M0 macrophages, M1 macrophages, M2 macrophages, and regulatory T cells. *SIDT1* expression, in contrast, exhibited the opposite correlations with all of these immune cell subsets. Additionally, our findings strongly underline the significant role of seven PCD-related genes in immune infiltration in TB and indicate that Tfh cells, CD8+ T cells, neutrophils, activated DCs, monocytes, M0 macrophages, M1 macrophages, M2 macrophages, and regulatory T cells are factors related to the cumulative rate of TB.

**Figure 8:**
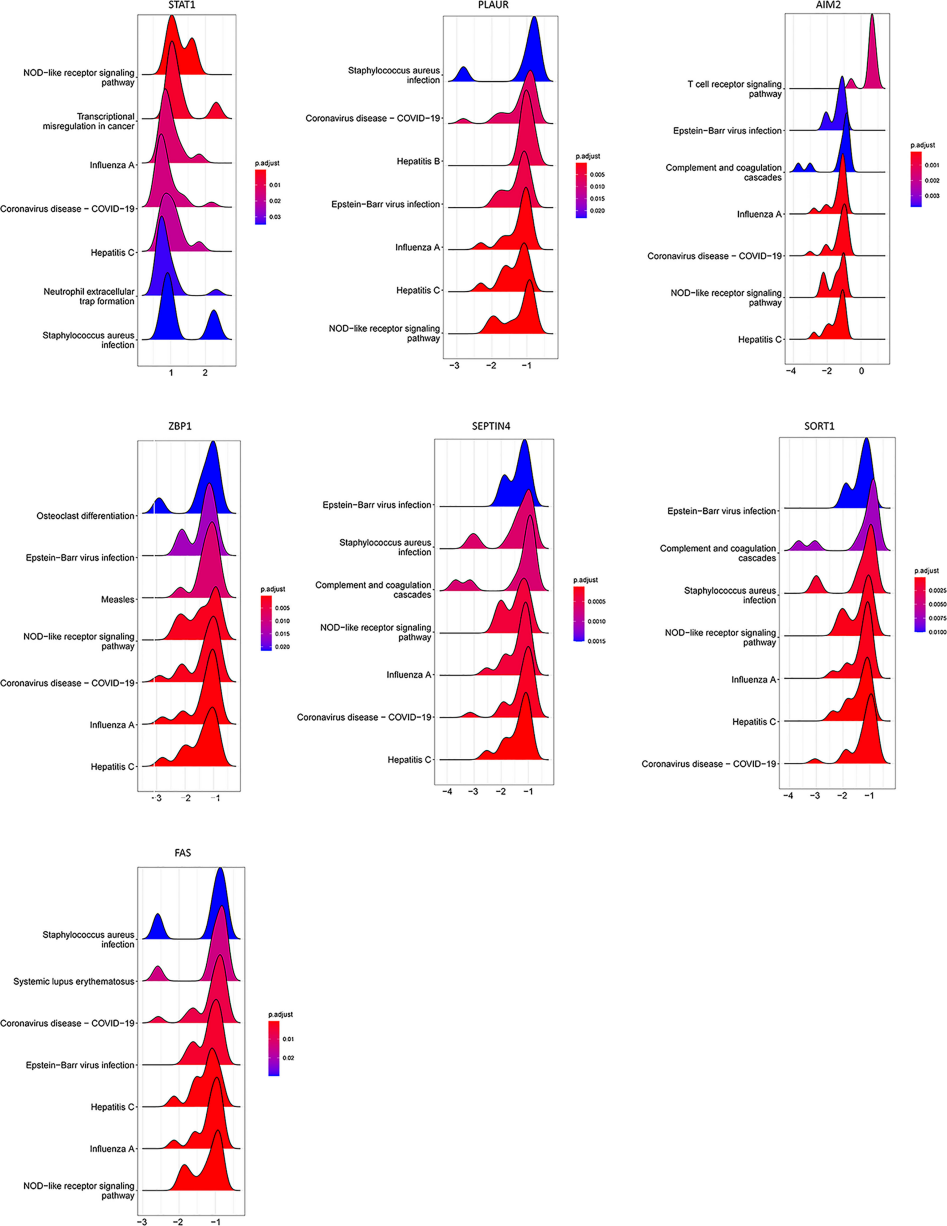
GSEA for samples with high and low PCD-related gene expression expression. The top seven enriched pathways in the high-low groups are shown.

Consistently, GSEA approaches indicated that these seven PCD-related genes were enriched in pathways associated with viral infection and immune-related activity (**Figure 8**).

### PCD-related gene signature-based consensus clustering analyses

**Figure 9:**
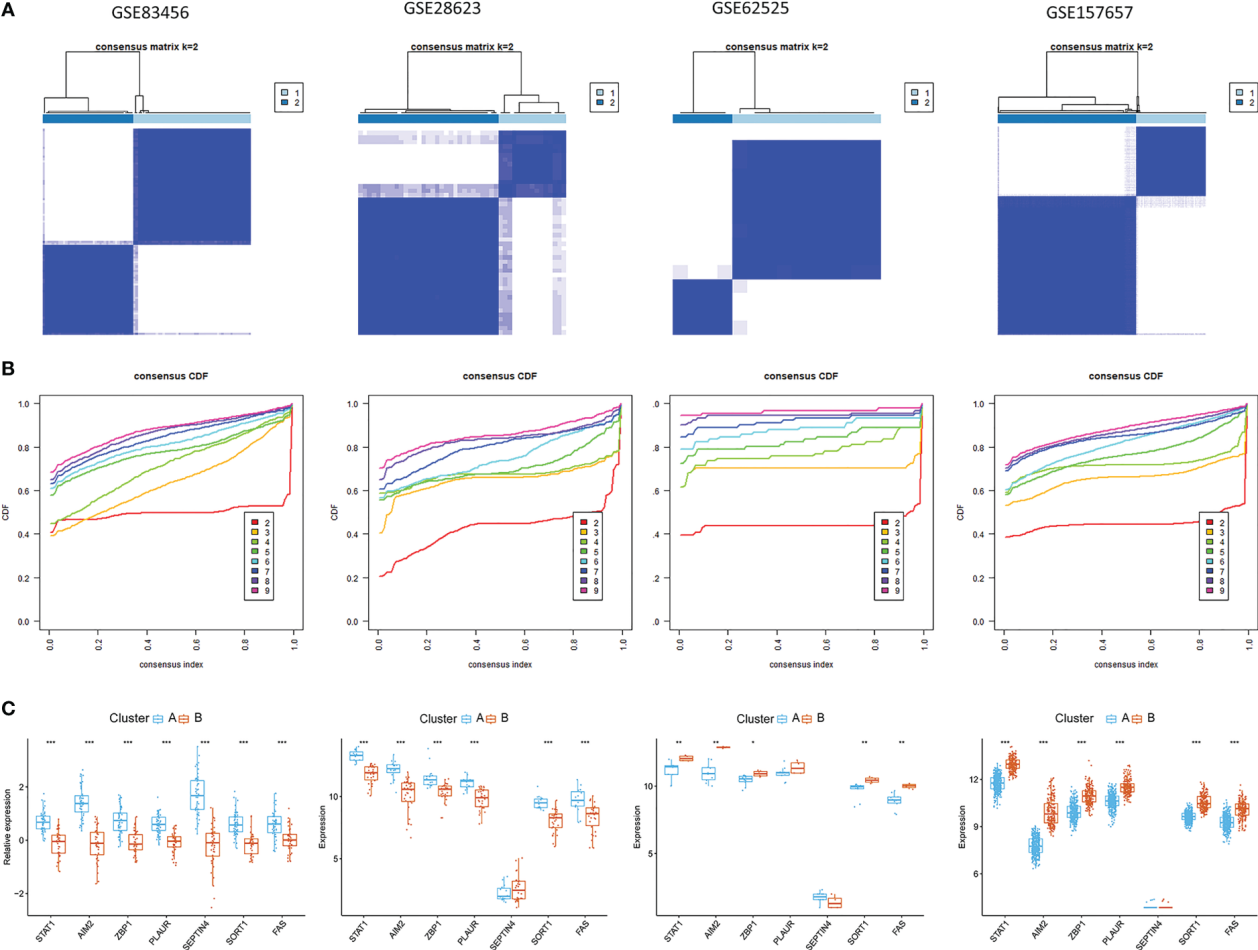
Identification of PCD-related subtypes in TB. **(A, B)** Subclustering analyses were performed based on differentially expressed genes in different datasets. **(C)** Differential PCD-related gene expression in the two established PCD subtypes.

To identify novel TB patient subgroups, these seven hub PCD-related genes were utilized for a consensus clustering analysis. At a k-value of 2, TB samples were effectively divided into two distinct clusters (**Figures 9A, B**), revealing significant differences between these two groups with respect to gene expression patterns (**Figure 9C**). Similar findings were also evident in the GSE28623, GSE62525, and GSE157657 datasets.

### Gene set variation analyses of PCD-based patient subsets

**Figure 10:**
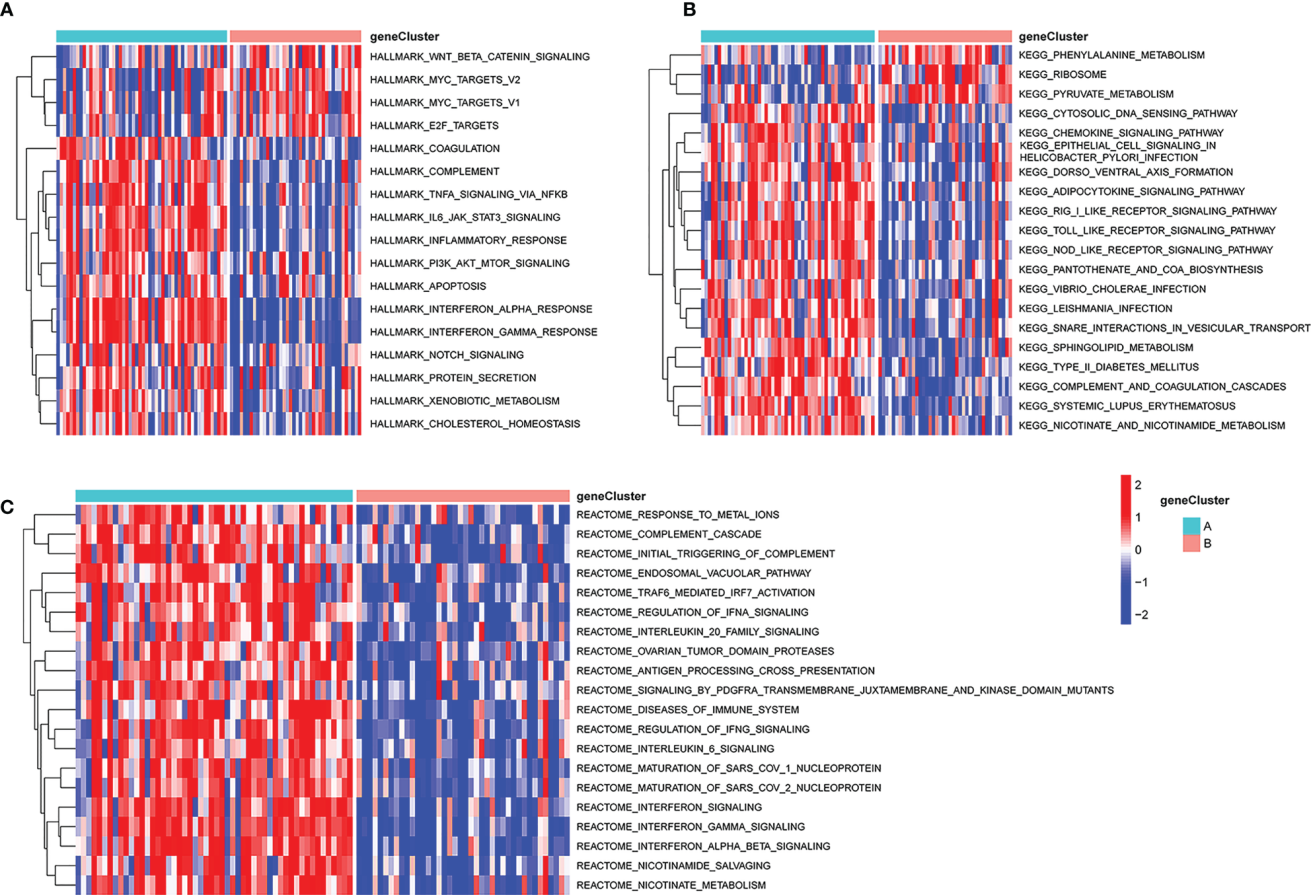
GSVA analysis of key pathways in different PCD-related patient subgroups. **(A)** HALLMARK pathway enrichment results. **(B)** KEGG pathway enrichment results. **(C)** Reactome pathway enrichment results. GSVA, gene set variation analysis.

The distinct biological processes active in these two subsets of patients were next examined via a GSVA approach. This approach revealed that the phenylalanine metabolism, ribosome, and pyruvate metabolism KEGG pathways were enriched in subgroup B, whereas the cytosolic DNA sensing and chemokine signaling pathways were enriched in subgroup A (**Figure 10B**). Relative to cluster A, the Hallmark Wnt/β-catenin signaling, MYC targets v2, MYC targets v1, and E2F targets pathways were significantly enriched in subgroup B, whereas the coagulation, complement, and TNF-α signaling via NFκB pathways were enriched in subgroup B (**Figure 10A**). Reactome pathway analyses also indicated that the top 20 pathways were more enriched in subgroup A relative to subgroup B (**Figure 10C**).

### Functional and immune signature-related analyses of the established PCD-related gene signature

**Figure 11:**
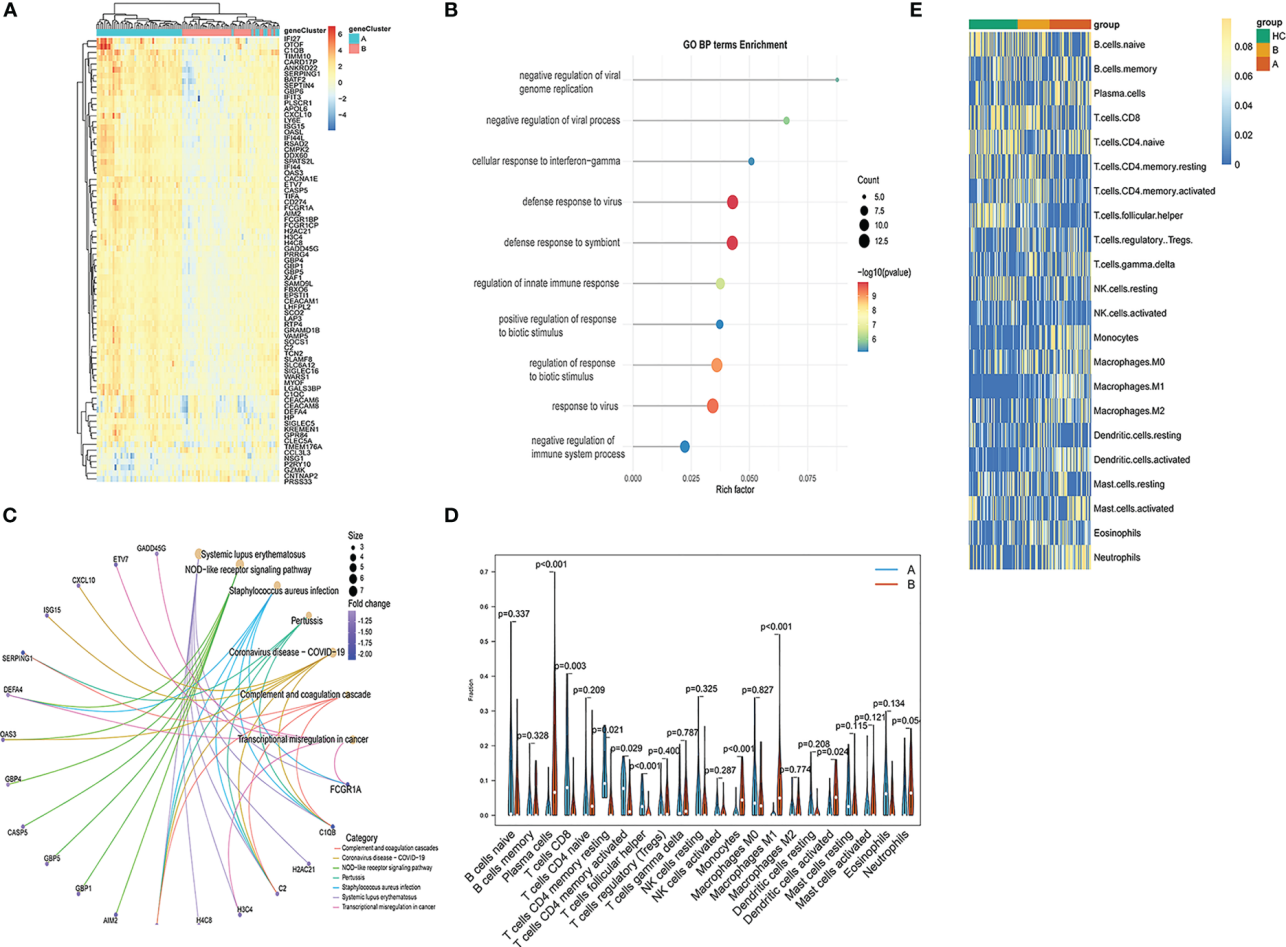
Functional enrichment analysis and immune cell enrichment analyses in different PCD-related patient subgroups. **(A)** DEGs arranged in volcano plots. **(B)** Enriched GO terms. **(C)** Enriched KEGG pathway analysis results. **(D)** Correlation matrix of all 22 immune cell subtype compositions. **(E)** Heatmap showing the relationship between gene expression levels and immune cell enrichment.

Functional differences between these two identified subgroups of TB patients were further examined through comparisons of gene expression patterns. Relative to subgroup A, subgroup B exhibited 77 DEGs of which 71 and 6 were respectively down- and upregulated (**Figure 11A**). These DEGs were enriched in GO BP terms including defense response to virus, innate immune response, response to virus, negative regulation of viral genome replication, etc. (**Figure 11B**), as well as in the systemic lupus erythematosus, NOD-like receptor signaling, Staphylococcus aureus infection, coronavirus disease-COVID-19, and pertussis KEGG pathways (**Figure 11C**). CIBERSORT analyses of immune cell enrichment revealed that relative to subgroup B, samples in subgroup A exhibited a higher proportion of CD8+ T, CD4+ memory activated T, CD4+ memory resting T and Tfh cells together with lower proportions of monocytes, M1 macrophages, activated DCs, and neutrophils (**Figure 11D**). Associations between PCD-related gene expression and immune cells were further examined, revealing positive correlations between PCD-related gene expression and neutrophils, activated mast cells, and M1 macrophages, as well as negative correlations between these genes and naïve CD4+ T cells, CD8+ T cells, resting memory CD4+ T cells, and Tfh cells (**Figure 11E**).

### PCD-related signature-based subclustering of other TB-related diseases

**Figure 12:**
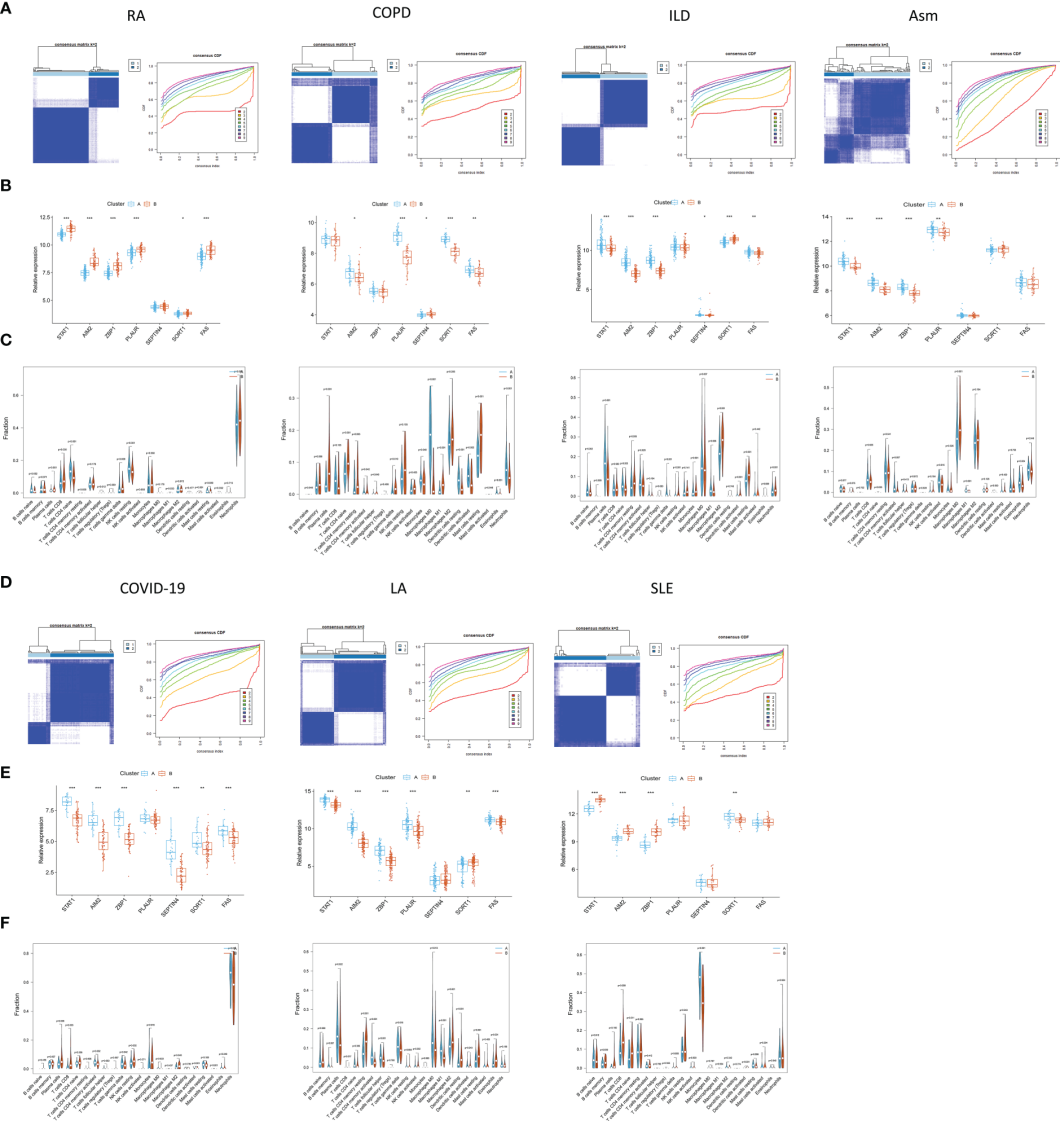
Identification of PCD-related subtypes in TB-related diseases. **(A, B, D, E)** Subclustering analyses were performed based on differentially expressed genes in different diseases. **(C, F)** PCD-related gene expression in different subtypes.

The pathogenesis of TB shares many characteristics with a range of other diseases including rheumatoid arthritis (RA), chronic obstructive pulmonary disease (COPD) (44), interstitial lung disease (ILD), asthma (Asm) (45), COVID-19 (46, 47), lung adenocarcinoma (LA) (48–50), and systemic lupus erythematosus (SLE) (51). Accordingly, subclustering analyses were performed based on the PCD-related signature established above, revealing that at a k-value of 2, patients for all diseases other than Asm were clearly stratified into two clusters (**Figure 12**). Clear differences in the expression of most PCD-related genes were observed when comparing these two patient subgroups in the analyzed diseases.

**Figure 13:**
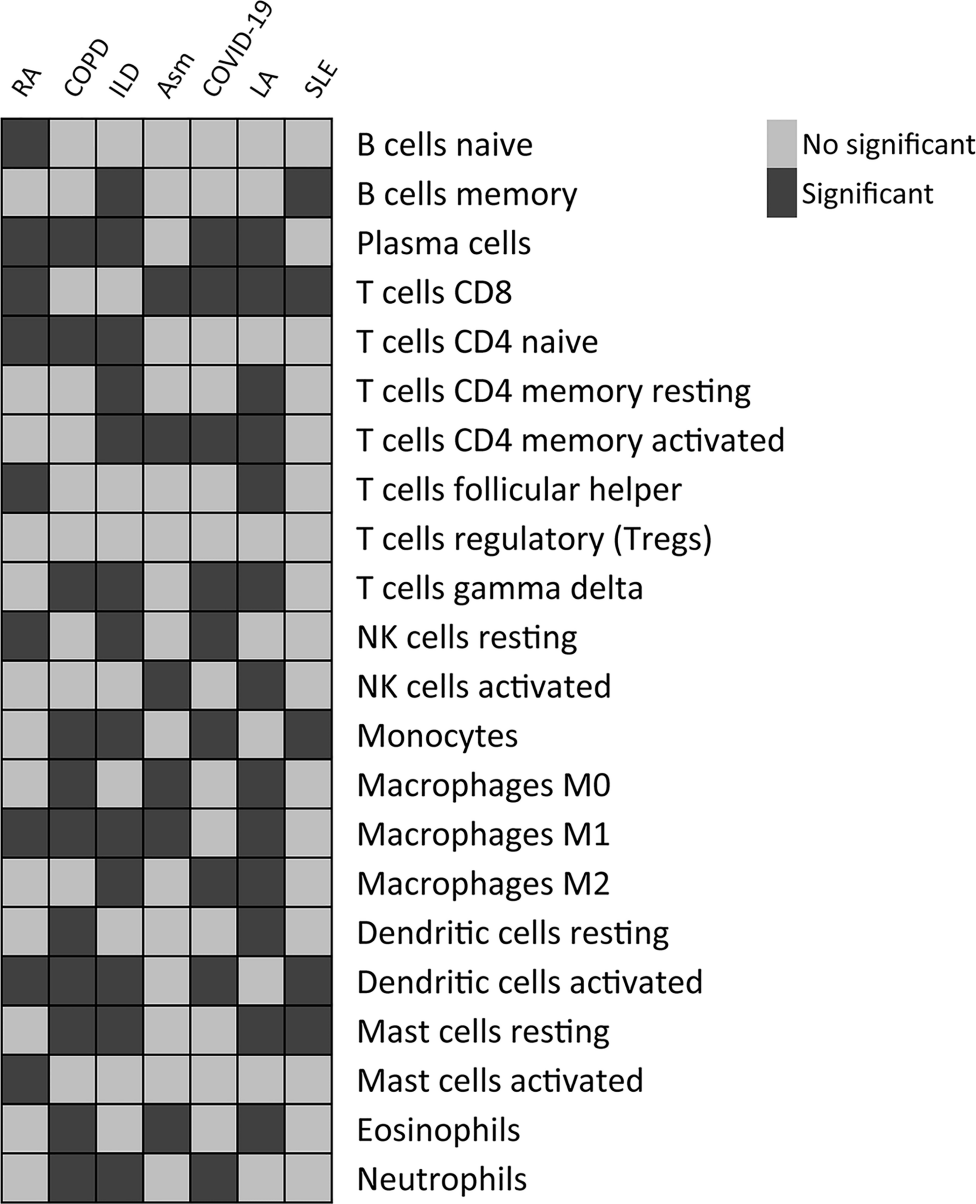
Differences in the relative enrichment of 22 different immune cell subtypes in TB-related disease subclusters.

The CIBERSORT algorithm was further used to evaluate the enrichment of 22 immune cell types in these diseases, revealing significant differences in immune cell enrichment when comparing subgroups A and B. Specifically, there were significant differences in the abundance of plasma cells, CD8+ T cells, M1 macrophages, and activated DCs in cluster A relative to cluster B in these five diseases, whereas no differences in regulatory T cell enrichment were observed (**Figure 13**). These findings thus confirmed the broader relevance of this PCD-related gene signature in different pathological settings.

### Verification of hub PCD-related genes expression by qPCR

**Figure 14:**
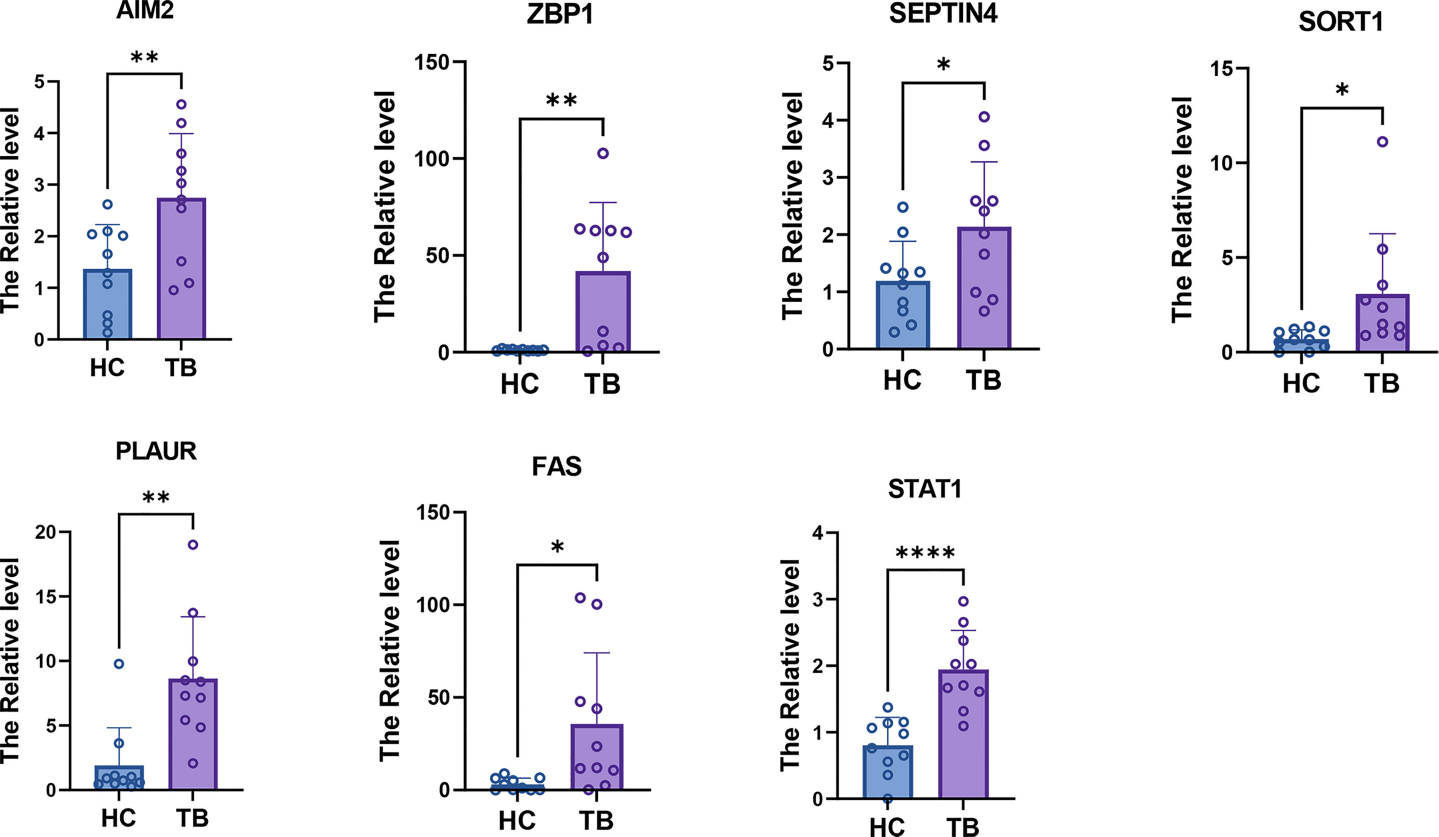
qPCR results showed that the expression levels of seven hub PCD-related genes. * = p < 0.05; **=p<0.01; **** = p < 0.001.

The expression of the seven hub PCD-related genes was then verified in TB plasma samples by qPCR. Consistent with the prediction, the results showed that the expression levels of the hub genes in the plasma of TB patients were significantly higher than those of the HCs (**Figure 14**).

### CMap predicted potential therapeutic agent for patients with TB-related diseases

**Figure 15:**
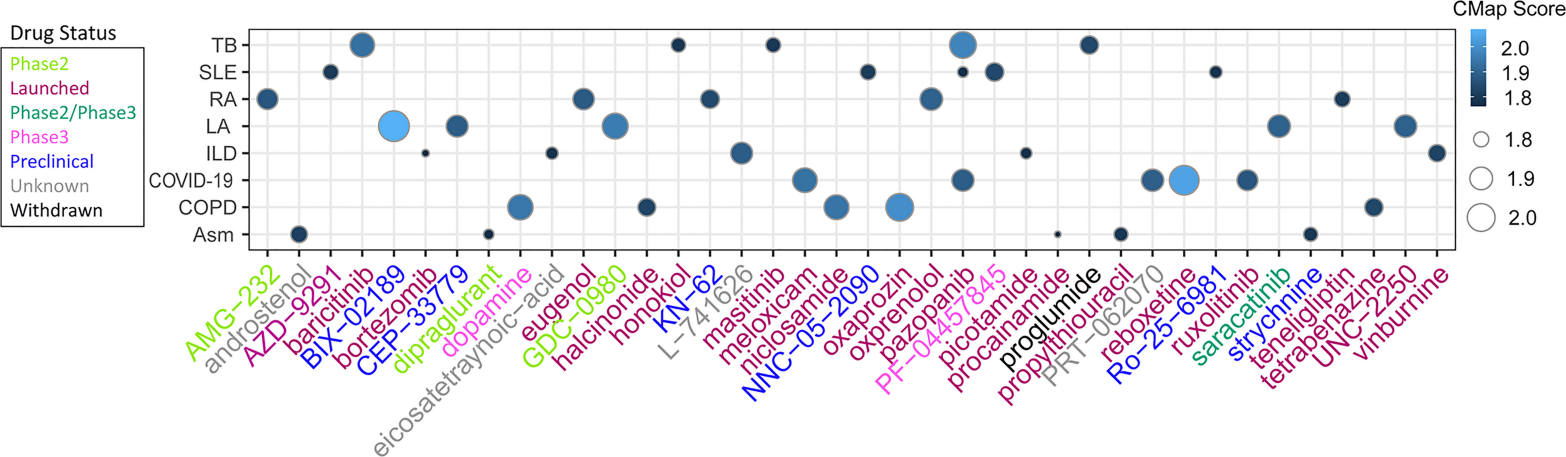
Heatmap showing CMap scores of PCD-related DEGs to drugs. The size of dots corresponds to absolute values of the CMap score.

To investigate potential drugs for high-risk patients with TB -related diseases, the anti-disease small molecule compounds were predicted by CMap analysis. As shown in **Figure 15**, these drugs Score higher and they were TB -related diseases inhibitor. The results showed that they might have an intervening effect on TB-related diseases progression (**Figure 15**).

## Discussion

TB is the deadliest infectious disease in the world, and patient morbidity and mortality continue to rise (52). However, the molecular drivers that contribute to negative TB patient outcomes remain incompletely understood. A range of drug combinations has been employed to treat TB over the last four decades, with treatment for a minimum of six months often being necessary. A 12-18 month treatment course is generally required for XDR-TB patients, achieving positive outcomes in just half of cases and subjecting patients and their families to serious economic hardship (53). These anti-TB drugs are also limited by their interactions with other drugs and their potential to cause aberrant inflammatory reactions in treated hosts that can result in permanent lung tissue damage (54). Novel treatment strategies are thus warranted to treat TB patients and to design novel drugs that can target particular pathways linked to the pathophysiological basis of TB. Accordingly, this study was designed to explore the relationship between PCD activity and TB and to better define key genes and therapeutic target candidates through a series of bioinformatics analyses.

Comparisons of gene expression patterns in TB patients and healthy controls led to the selection of 149 DEGs, and GO enrichment analyses indicated that these genes were primarily enriched in the defense response to virus, internal immune response, response to virus 17, negative regulation of viral genome replication, interleukin-27 mediated signaling pathway, and immune response GO term categories. Innate immune activity plays an important role in shaping pathogenic bacterial elimination and inducing adaptive immune responses mediated by central memory and effector T cells) (55). IL-27 is a cytokine that exhibits a range pro- and anti-inflammatory properties and thereby acts as a key mediator of bacterial infection-related immune responses (56).

Immune cell enrichment analysis demonstrated that T cells gamma delta, monocytes, M0, M1, and M2 macrophages, activated DCs and neutrophils had higher proportions in TB samples. Mtb diversifies its niche by infecting neutrophils, DCs, and macrophage groups resident and recruited by various tissues; Neutrophils create a good environment for the replication of Mtb, and the disease progress is closely related (2, 57–59). Both CD4+T and CD8+T cells have been found to have protective effects against Mtb infection and the lower proportion of them make patient was easy to be infected by Mtb (60, 61). Except for the results of DCs and navie CD4+ cells, almost all the results were consistent with the results of single-cell sequencing analysis. This further demonstrates the importance of immunity in the development of TB.

PCD is an umbrella term that refers to a variety of complex, interrelated processes governed by a range of mechanisms. PCD activities have increasingly been shown to be linked to a variety of disease states (11, 22, 23, 62). As such, the association between PCD-related genes and TB phenotypes was herein examined, with multiple machine learning algorithms being used to establish a predictive signature comprised of seven PCD-associated genes (*STAT1*, *AIM2*, *ZBP1*, *PLAUR*, *SEPTIN4*, S*ORT1*, *FAS*) that exhibited excellent diagnostic utility for TB. STAT1 serves as a central mediator of interferon signaling and the induction of anti-TB immune responses (63), with higher levels of unphosphorylated STAT1 reportedly decreasing macrophage sensitivity to apoptotic death induced by FAS in the context of Mtb infection (64). The cytosolic sensor protein AIM2 can detect the dsDNA released by damaged cells, whereupon it induces the upregulation and secretion of various cytokines such that it drives the pathogenesis of various inflammatory diseases (65). ZBP1 is capable of binding RIPK3 and activates it to induce necrotic death (66), while also complexing with pyrin and AIM2 to coordinate host defense responses (67). The SEPTIN3 protein has previously been shown to be ectopically expressed in TB, colorectal cancer, and urologic cancers such that it can be leveraged as a valuable diagnostic biomarker (28, 68). The three-domain PLAUR protein is capable of binding to cell membranes via glycolipid anchor motifs, and exhibits a range of regulatory roles in particular pathological settings (69, 70). The sortilin protein encoded by SORT regulates LDL uptake in addition to having been used as a biomarker in several forms of disease (71, 72). The results of the enrichment analysis also indicated a possible association between the seven hub genes and immunity. Thus, correlations between these genes and immune cell levels were also analyzed. *SORT1* has been shown to promote the proliferation and migration of liver cancer cells by regulating immune cell infiltration (73). *AIM2*, *ZBP1*, *STAT1*, and *FAS* mediate the immune response and play important roles in a variety of diseases, cancers, and infections (65, 74–76). Although there is no clear research indicating their role in immune cell infiltration in TB, changes in their levels have been shown to have an impact on immune cell levels. Relative to HC samples, these genes were upregulated in samples from individuals with TB, emphasizing potentially key roles for these PCD-associated genes as mediators of TB pathogenesis. However, further direct experimental validation of this hypothesis will be required.

Based on these seven PCD-related genes, consensus clustering was used to define two PCD-related clusters, with all seven of these hub PCD-related genes being upregulated in subgroup A relative to subgroup B. Subgroup A exhibited immune-related gene enrichment in a GSVA analysis, and identified DEGs were found to be enriched in the response to virus and innate immune response GO terms. Samples from individuals in subgroup A also exhibited enriched plasma cells, M1 macrophages, and activated DCs. Macrophages are important innate immune cells that can detect Mtb-derived pathogen-associated molecular patterns through the Toll-like, NOD-like, and C-type lectin receptor pathways and the cGAS-STING pathway, enabling these cells to rapidly react to these mycobacteria by engaging an appropriate immune response (2). M1 macrophages, in particular, are important mediators of pro-inflammatory responses (77). In this study, a positive correlation between PCD-related gene expression and proinflammatory response activity was detected. However, the M2/M1 proportion in Subgroup A was reduced, in contrast to prior reports suggesting a link between PCD activity and anti-inflammatory activity (24). Mycobacterial antigens are transferred to DCs, which can then present these antigens on MHC class I molecules to CD8+ T cells (78). Both CD4+ and CD8+ T cells, in turn, coordinate anti-mycobacterial immunity (79). The potential drugs predicted by CMAP based on the high- and low- PCD-related groups may be used to treat these TB-related diseases. In summary, the PCD-related genes identified in the present study offer value as diagnostic indicators and possible therapeutic targets in both TB and a range of other diseases.

There are some limitations to this analysis. For one, these results remain to be validated through further experimental analyses and clinical trials. Moreover, these results were derived from large public databases and the original sequencing data were unavailable, potentially introducing some degree of selection bias. The sample sizes were also relatively small such that additional TB patients will be required to confirm these findings. Functional verification of the importance of these PCD-related genes in TB will also be important.

## Conclusion

In summary, these results highlight a clear relationship between PCD-related gene expression and immune cell infiltration in TB, while also revealing differences in the immune responses engaged in different PCD-related gene-based TB patient subgroups. The use of machine learning models enabled the effective selection of the optimal PCD-related genes capable of evaluating TB patient subtypes and guiding the diagnosis of this disease. Accordingly, these results offer novel evidence in support of the involvement of these PCD-associated genes in the progression of TB while offering new insights regarding the pathogenic basis for this disease and potential approaches to improving infected patient outcomes.

## Data availability statement

The original contributions presented in the study are included in the article/**Supplementary Material**. Further inquiries can be directed to the corresponding author.

## Ethics statement

This study was approved by the institutional review board and all patients gave written informed consent.

## Author contributions

Conceptualization, JS and ZL. Methodology, HZ and PZ. software, JS, CZ. Validation, JS, CZ, HZ. resources, ZL. data curation, JS, CZ, HZ. writing—original draft preparation, JS and ZL. writing—review and editing, HZ. visualization, ZL. supervision, ZL. project administration, ZL. funding acquisition, ZL. All authors contributed to the article and approved the submitted version.
