# Correction: Classification of tuberculosis-related programmed cell death-related patient subgroups and associated immune cell profiling

**DOI:** 10.3389/fimmu.2026.1901643

**Published:** 2026-07-10

**Authors:** Jie Shen, Chao Zhao, Hong Zhang, Peipei Zhou, Zhenpeng Li

**Affiliations:** 1 School of Medical Laboratory, Weifang Medical University, Weifang, China; 2 Office of Academic Affairs, Weifang Medical University, Weifang, China; 3 School of Public Health, Weifang Medical University, Weifang, China

**Keywords:** programmed cell death, tuberculosis, immune cell enrichment, single cell RNA-seq, machine learning, biomarkers

File **Supplementary Table 2** was erroneously published with the original version of this paper. The file has now been replaced.

Furthermore, in the published article, there was a mistake in the **Materials and methods** section, subsection “*Quantitative reverse transcription polymerase chain reaction*”, paragraph 2 (starting with “Samples were collected from each participant…”).

The original text incorrectly stated that “U6 served as an internal control.” This was a clerical error during manuscript preparation. In fact, all qPCR experiments were performed using GAPDH as the internal control, and the quantitative data were properly normalized to GAPDH. The authors sincerely apologize for this mistake.

We confirm that all experimental procedures, data analyses, and research conclusions relied on GAPDH normalization from the very beginning of the study. Consequently, this textual correction has no bearing whatsoever on any results or conclusions presented in the article.

A correction has been made to the section **Materials and methods**, “*Quantitative reverse transcription polymerase chain reaction*”, Paragraph 2: “GAPDH served as an internal control”

The original version of this article has been updated.

## Figures and Tables

**Table S2:** Oligonucleotides used in this study.

Name	Up primer	Down primer
FAS	TCTGTCTGCTGTGTCTTGGA	TCACCACTATTGCTGGAGTCA
ZBP1	CGAGACATTGGTCAGCCCAT	CTGCCTTCTGCAGCTTTGTG
SORT1	AAGTCTTTGGACCGACATCTCT	AGCACGCTTGTTATGTAGACG
STAT1	GCAGCTCATTCAGAGCTCGTT	TCATTCACATCTCTCAACTTCACA
SEPTIN4	AGGACTGAAGCTGGGATCAA	CAGCTCGCATTTCCTGAGAA
PLAUR	GAGCTATCGGACTGGCTTGAA	CGGCTTCGGGAATAGGTGAC
GAPDH	GATTCCACCCATGGCAAATTC	CTGGAAGATGGTGATGGGATT
AIM2	CGTGCTGCACCAAAGTCTC	GGCAAACAGCGCTTCTGAAA

