# Supplementary material for: Classification of tuberculosis-related programmed cell death-related patient subgroups and associated immune cell profiling
Source: Front Immunol. 2023 May 2;14:1159713. doi: 10.3389/fimmu.2023.1159713 (PMC10185908; doi:10.3389/fimmu.2023.1159713)
Supplement: Supplementary file 2 [file Table_2.docx]

Table S2 Oligonucleotides used in this study

| Name | Up primer | Down primer |
| --- | --- | --- |
| FAS | TCTGTTCTGCTGTGTCTTGGA | TCACCACTATTGCTGGAGTCA |
| ZBP1 | CGAGACATTGGTCAGCCCAT | CTGCCTTCTGCAGCTTTGTG |
| SORT1 | AAGTCTTTGGACCGACATCTCT | AGCACGCTTGTTATGTAGACG |
| STAT1 | GCAGCTCATTCAGAGCTCGTT | TCATTCACATCTCTCAACTTCACA |
| SEPTIN4 | AGGACTGAAGCTGGGATCAA | CAGCTCGCATTTCCTGAGAA |
| PLAUR | GAGCTATCGGACTGGCTTGAA | CGGCTTCGGGAATAGGTGAC |
| GAPDH | GATTCCACCCATGGCAAATTC | CTGGAAGATGGTGATGGGATT |
| AIM2 | CGTGCTGCACCAAAAGTCTC | GGCAAACAGCGCTTCTGAAA |
