# Supplementary material for: Classification of tuberculosis-related programmed cell death-related patient subgroups and associated immune cell profiling
Source: Front Immunol. 2023 May 2;14:1159713. doi: 10.3389/fimmu.2023.1159713 (PMC10185908; doi:10.3389/fimmu.2023.1159713)
Supplement: Supplementary file 3 [file DataSheet_1.zip › Supplementary Figure/Supplementary Figure legend.docx]

Figure. S1 (A) Violin plots of classical markers for T, B cells and myeloid. (B) Dot plot of marker genes for distinct cell types. Dot size is proportional to the number of cells in each cluster that express the marker genes, and color scale indicates normalized expression of marker genes.

Figure. S2 (A) Violin plots of classical markers for myeloid subclusters. (B) Dot plot of marker genes for myeloid subclusters.

Figure. S3 (A) Violin plots of classical markers for T subclusters. (B) Dot plot of marker genes for T cells subclusters.

Figure. S4 (A) UMAP visualization of major cell types across three groups (left), Myeloid clusters (middle), and T cells (right) based on SRR11038990 and SRR11038994 datasets. (B) Proportion of each defined cell type across major groups (left), myeloid clusters (middle), and T cells (right) based on SRR11038990 and SRR11038994 datasets. B, B cells; T, T cells; M, Myeloid. NK, Naturel killer
