# Supplementary figures and images for: Classification of tuberculosis-related programmed cell death-related patient subgroups and associated immune cell profiling
Source: Front Immunol. 2023 May 2;14:1159713. doi: 10.3389/fimmu.2023.1159713 (PMC10185908; doi:10.3389/fimmu.2023.1159713)

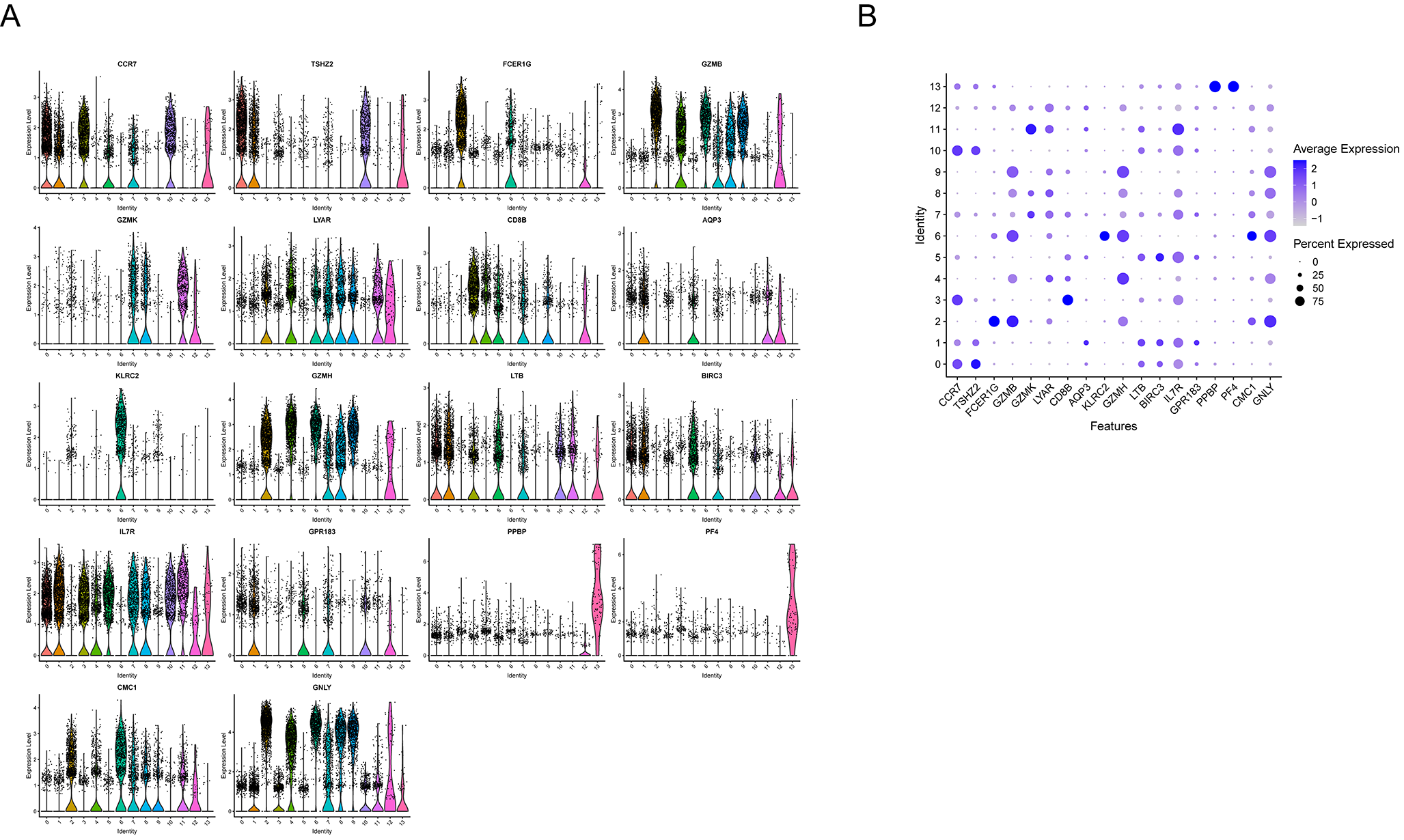

Supplement: Supplementary file 3 [file DataSheet_1.zip › Supplementary Figure/Figure S3.tif]
